# Yin/Yang expression of CCN family members: Transforming growth factor beta 1, via ALK5/FAK/MEK, induces CCN1 and CCN2, yet suppresses CCN3, expression in human dermal fibroblasts

**DOI:** 10.1371/journal.pone.0218178

**Published:** 2019-06-06

**Authors:** Alexander Peidl, Bernard Perbal, Andrew Leask

**Affiliations:** 1 Department of Physiology and Pharmacology, University of Western Ontario, London, ON, Canada; 2 Université Côte d’Azur, CNRS, GREDEG, Nice, France; 3 Department of Dentistry, University of Western Ontario, London, ON, Canada; Boston University Henry M Goldman School of Dental Medicine, UNITED STATES

## Abstract

The role of the microenvironment in driving connective tissue disease is being increasingly appreciated. Matricellular proteins of the CCN family are signaling modifiers that are secreted by cells into the extracellular matrix microenvironment where they have profound, context-dependent effects on organ development, homeostasis and disease. Indeed, CCN proteins are emergent targets for therapeutic intervention. Recent evidence suggests that, in vivo, CCN3 has effects opposing CCN2. Moreover, when CCN3 expression is high, CCN2 expression is low. That is, they appear to be regulated in a yin/yang fashion, leading to the hypothesis that the CCN2:CCN3 ratio is important to control tissue homeostasis. To begin to test the hypothesis that alterations in CCN2:CCN3 expression might be important in skin biology in vivo, we evaluated the relative ex vivo effects of the profibrotic protein TGFbeta1 on dermal fibroblasts on protein and RNA expression of CCN3 and CCN2, as well as the related protein CCN1. We also used signal transduction inhibitors to begin to identify the signal transduction pathways controlling the ability of fibroblasts to respond to TGFbeta1. As anticipated, CCN1 and CCN2 protein and mRNA were induced by TGFbeta1 in human dermal fibroblasts. This induction was blocked by TAK1, FAK, YAP1 and MEK inhibition. Conversely, TGFbeta1 suppressed CCN3 mRNA expression in a fashion insensitive to FAK, MEK, TAK1 or YAP1 inhibition. Unexpectedly, CCN3 protein was not detected in human dermal fibroblasts basally. These data suggest that, in dermal fibroblasts, the profibrotic protein TGFbeta1 has a divergent effect on CCN3 relative to CCN2 and CCN1, both at the mRNA and protein level. Given that the major source in skin in vivo of CCN proteins are fibroblasts, our data are consistent that alterations in CCN2/CCN1: CCN3 ratios in response to profibrotic agents such as TGFbeta1 may play a role in connective tissue pathologies including fibrosis.

## Introduction

Fibrosis, as a pathology, is characterized by excessive deposition of extracellular matrix, comprised principally of type I collagen, resulting in scar tissue that ultimately culminates in organ dysfunction and death. Collectively, fibrosis and fibrosis-associated disorders account for ~45% of the health care costs and deaths in the Western world [1]. As a feature of end-stage disease, the contribution of fibrosis to human disease would be expected to rise due to an increasingly aging population. Fibrotic conditions of the skin include: hypertrophic scars that occur in response to burns or wounding, keloids, or scleroderma, in which skin (and internal organs) progressively scars resulting in dermatological effects such as itching, skin tightness and reduced mobility [2,3].

The effector cell of fibrosis is the fibroblast, which responds to profibrotic cytokines such as TGFbeta by increasing production, contraction, adhesion and remodeling of the surrounding extracellular matrix [2, 4]. Initially it was believed, owing to its profound in vitro and in vivo effects and its potent upregulation in connective tissue disease, that targeting TGFbeta and its canonical signaling pathways would have profound palliative effects on fibrotic conditions. However, it is now widely appreciated due to its established pleiotropic effects, to not be an appropriate therapeutic target due to lack of efficacy relative to observed side effects [4,5]. This problem was surmised a priori, leading to the search in the early 1990s for downstream effectors or cofactors of TGFbeta that may have more selective profibrotic effects [6]. Indeed, parallel studies examining: (1) non-canonical TGFbeta signaling; (2) the mechanobiology of the profibrotic effector cell, the myofibroblast; and (3) collagen structure conclusively established that an enhanced, autocrine pro-adhesive signaling pathway was essential to promote and sustain fibrosis [7–11].

The convergence of these approaches, namely those involving the identification of possible cofactors/downstream mediators of TGFbeta and of an autocrine pro-adhesive signaling loop in promoting and sustaining fibrosis, have supported the hypothesis that targeting the cellular microenvironment may be an appropriate therapeutic approach [2, 12, 13]. In particular, the CCN family of secreted pro-adhesive matricellular proteins are of interest [14, 15]. CCN2 (formerly called CTGF), which is induced in fibroblasts by the potent profibrotic cytokine TGFbeta, was hypothesized as being a mediator of fibrosis as early as the mid-1990s [6, 16, 17]. Indeed, conditional knockout strategies have shown CCN2 expression by fibroblasts is required for fibrosis in a variety of mouse models [15, 18–21]. Conversely, CCN2 is not required for cutaneous tissue repair [22], emphasizing its selective profibrotic action and its potential utility as a specific anti-fibrotic target. Significantly, an anti-CCN2 antibody strategy (FG-3019) is currently entering a Phase III trial for idiopathic pulmonary fibrosis [23].

In addition to CCN2, CCN1 has context-specific profibrotic effects [24]. Thus, clinically, a more precise strategy might be to target both CCN1 and CCN2 simultaneously. In that regard, another member of the CCN family, CCN3, is reciprocally regulated by CCN2 in a model of diabetes [25,26], in glomerular cell proliferation [27], and chondrocyte differentiation [28]. Moreover, CCN3 protein has antifibrotic effects in a diabetes model [29]. These data have led to the hypothesis that a high CCN2:CCN3 ratio drives fibrosis and that normalizing this ratio by adding CCN3 may have antifibrotic effects [14, 30]. In addition, reciprocal regulation of CCN1 and CCN3 activities has also been previously discussed [31]. However, no studies have simultaneously examined the regulation of CCN1, CCN2, and CCN3, possibly because, until recently, the concept of all three proteins being members of the same family and therefore worthy of studying them simultaneously has not achieved widespread recognition [30, 32].

To begin to address this conceptual deficit, we elected to determine to add TGFbeta1 to human dermal fibroblasts and simultaneously monitor the expression of CCN1, CCN2 and CCN3. Moreover, we use chemical signal transduction inhibitors to identify if a common pathway mediates TGFbeta1’s effects on CCN1, CCN2 and CCN3. Our data provide new and valuable insights into the reciprocal regulation of CCN proteins in fibroblasts and into the signaling mechanisms downstream of TGFbeta1 in driving expression of key profibrotic mediators in fibroblasts.

## Methods

### Cell culture

All cell culture experiments were performed using primary human foreskin fibroblasts obtained from healthy humans (American Type Culture Collection CRL2094) that were previously shown to differentiate into myofibroblasts in the presence of TGFbeta 1 or mechanical tension [33–35]. Cells were cultured to passage 8 in high glucose DMEM (Invitrogen) supplemented with 10% FBS and 1% antibiotic-antimycotic solution. Cells were plated on tissue culture plastic at an approximate density of 60000 cells/plate and grown overnight at 37°C in a humidifier containing 5% CO_2_. At approximately 70% confluence, cells were serum starved by replacing high glucose DMEM with low glucose DMEM containing 0.5% FBS and 1% antibiotic-antimycotic solution. After serum starving overnight, cells were pre-treated with either DMSO or one of the following small molecule inhibitors: SB-431542 (Tocris, 10 μM), PF573228 (Sigma, 10 μM), (5Z)-7-oxozeaenol (Tocris, 400 μM), U0126 (Sigma, 30 μM), or Verteporfin (Sigma, 695 nM). TGFbeta1 (R&D Systems, 4 ng/ml) was added 30 minutes after inhibitors for either 6 hours (for RNA collection) or 24 hours (for protein collection).

### Generation of CCN3-overexpressing fibroblasts

Custom lentiviral particles were designed and obtained from Sigma-Aldrich. CCN3 overexpressing cells were generated using a CSTORFV Mission TRC3 Custom Human ORF Lentivirus (pLX317) containing a specifically designed CCN3 expression vector. Transduction control cells were generated using an ORFBFPV Mission TRC3 ORF GFP Lentivirus. For transduction, human foreskin fibroblasts were incubated with viral particles at a moiety of infection of 1.5 supplemented with 5 μg/mL Polybrene for 24 hours. Successfully transduced cells were selected for using puromycin as described by the manufacturer (Sigma-Aldrich). Surviving cells were cultured to passage 8.

### Real-time PCR

Total RNA was obtained from treated cells after 6 hour treatments described above. TriZol extraction using phenol-chloroform was used to isolate RNA from cell lysates to be used for real-time PCR. RNA concentrations and integrity were measured via Nanodrop 2000 (Thermo Scientific). A total of 1 μg of RNA from each sample was reverse transcribed using qScript Supermix (QuantaBio), producing cDNA. SYBR green real-time PCR was then performed by combining cDNA (7 ng/well), SYBR master mix (Thermo Scientific) and gene specific primers. Signal changes were detected using a ViiA 7 Real-Time PCR System (Thermo Scientific). The following gene specific primers were purchased from Life Technologies for use in our experiment: CCN1 Fw:5`-CGGCTCCCTGTTTTTGGAAT-3`, Rev: 5`-TTGAGCACTGGGACCATGAA-3`; CCN2 5`-GAGGAGTGGCTGTGTGACG-3`, 5`-TCTTCCAGTCGGTAACCGC-3`; CCN3 5`-GTGCTACTGCCTGAGCCTAA-3`, 5`-CTGTAAGCTGCAAGGGTAAGG-3`; EDN1 5`-AGAAACAGTCTTAGGCGCTGA-3`, 5`- TGGACTGGGAGTGGGTTTCT-3`; ITGA11 5`-CTGTGGCCAGGGTTCACG-3`, 5`-TGTAGCCAAAGAAGGCGGTC-3`; β-actin 5`-CCTCGCCTTTGCCGATCC-3`, 5`-CGCGGCGATATCATCATCG-3`. Samples were run in triplicate and expression values were standardized to control values from β-actin primers using the ΔΔCt method. Biological repeats are indicated. GraphPad Prism software was used to perform a one-way ANOVA with Tukey’s post-hoc test to determine statistical significance.

### Western blot

Proteins were harvested using radioimmunoprecipitation assay (RIPA) buffer (150 mM NaCl, 100 mM Tris-HCl pH 7.4, 1% NP40, 0.1% SDS, 5 mM EDTA, 1X protease inhibitor cocktail) from cells after 24 hour treatment. Protein concentrations were approximated using a BCA protein assay kit (Thermo Scientific), according to instructions provided by the distributor. An equal amount of protein (50 μg) was added to each well of an SDS-PAGE polyacrylamide gel (5% stacking, 10% separating). Protein samples were resolved and then transferred to a nitrocellulose membrane. Membranes were blocked in 5% non-fat skim milk in TBST (100mM Tris-HCl, pH 7.4, 0.01% Tween-20) for 1 hour, and then incubated with primary antibody overnight at 4°C in 5% milk solution. The following antibodies were used: anti-CCN1 (1:1000; abc102; Santa Cruz), anti-CCN2 (1:500; sc14939; Santa Cruz) and anti-beta-actin (1:8000; A1978; Sigma-Aldrich). Anti-CCN3 antibody (dilution 1:2000), was used as described in the paper disclosing the generation of the antibody [36]. Membranes were washed thoroughly in TBST and then incubated with HRP-conjugated secondary antibody for 1 hour. Horseradish peroxidase-conjugated donkey anti-goat (705-036-147), donkey anti-rabbit (711-036-152) and donkey anti-mouse (715-035-150) were obtained from Jackson Immunoresearch Laboratories. Membranes were washed thoroughly and then exposed to SuperSignal West Pico Chemiluminescent Substrate (Thermo Scientific) for 5 minutes before visualization using X-ray film.

## Results

### TGFbeta1 induces CCN1 and CCN2 yet suppresses CCN3 expression in human dermal fibroblasts via ALK5

**Fig 1:**
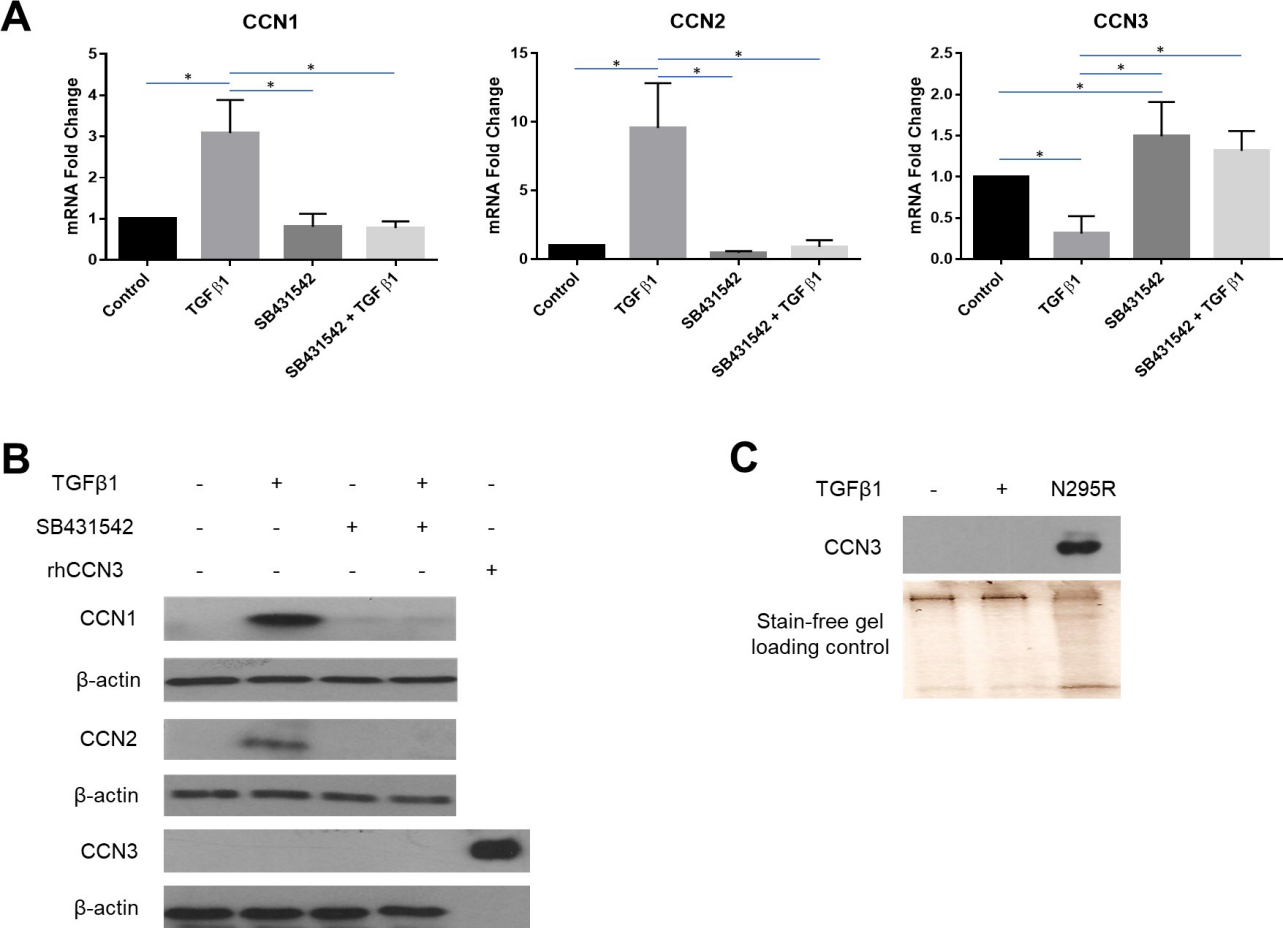
TGFbeta1-responsiveness of CCN1, CCN2 and CCN3 is sensitive to ALK5 inhibition in human dermal fibroblasts. Human dermal fibroblasts were cultured, and serum starved overnight, prior to pre-treatment with an ALK5 inhibitor, SB431542 (10 μM). After pre-treatment, cells were incubated either with or without TGFbeta1 (4 ng/ml). **(A)** Total RNA was extracted 6 hours after treatment and subjected to real-time PCR to detect changes in CCN1, CCN2 and CCN3 gene expression. Results are expressed as mean +/- SD. Statistical differences were determined using one-way ANOVA with Tukey’s post-hoc test (p<0.05, n = 6). **(B)** Protein was extracted 24 hours after treatment and equal amounts of protein (50 μg) were resolved using SDS-PAGE. Western blot analysis was performed using antibodies against CCN1, CCN2 and CCN3. Beta-actin was used as a loading control. Representative blots are shown (n = 3). **(C)** CCN3 protein is readily detected basally in NCI-H295R cells but not in human dermal fibroblasts.

To begin to assess if, in human dermal fibroblasts, TGFbeta1 affects CCN1, CCN2 and CCN3 expression in a yin/yang fashion, we treated serum-starved cells overnight and then cultured them with or without TGFbeta1 for an additional 6 h (for RNA analysis) or 24 h (for protein an analysis). RNA and protein were harvested. For our protein analysis, consistent with previous studies, we examined total cell extracts to capture CCN proteins in the process of being secreted through the Golgi. In our initial experiments, as a control, we also treated cells in the presence or absence of ALK5 inhibitor, as ALK5 is the TGFbeta type I receptor for fibroblasts [4]. As expected, TGFbeta1 induced CCN1 and CCN2 mRNA and protein, consistent with prior studies [16, 37] (Fig 1). Moreover, consistent with our prior studies, ALK5 inhibition blocked TGFbeta1 induced CCN1 mRNA and protein in human dermal fibroblasts [38] (Fig 1). Furthermore, TGFbeta1 also induced CCN2 mRNA protein in a fashion sensitive to ALK5 inhibition; conversely, TGFbeta1 suppressed CCN3 mRNA expression; this was also blocked by addition of ALK5 inhibitor (Fig 1).

However, to our surprise, CCN3 protein was undetectable in human dermal fibroblasts either in the presence or absence of added TGFbeta1 (Fig 1). CCN3 was readily detected NCI-H295R cells, a tumor line that expresses CCN3 [33], indicating the validity of our method of detecting CCN3 protein (Fig 1). That is, CCN3 does not appear to be basally expressed by proliferating human dermal fibroblasts (Previous studies have shown that low levels of intracellular CCN3 are produced in fibroblasts in culture; in growing cells the expression of CCN3 protein is quickly downregulated [37]). Collectively however, these data are consistent with the idea that, at least at the mRNA level, TGFbeta1 has opposing effects on CCN1 and CCN2 as compared to CCN3 in human dermal fibroblasts and that this effect is mediated by ALK5.

### TGFbeta1 induces CCN1 and CCN2 expression in human dermal fibroblasts via FAK

**Fig 2:**
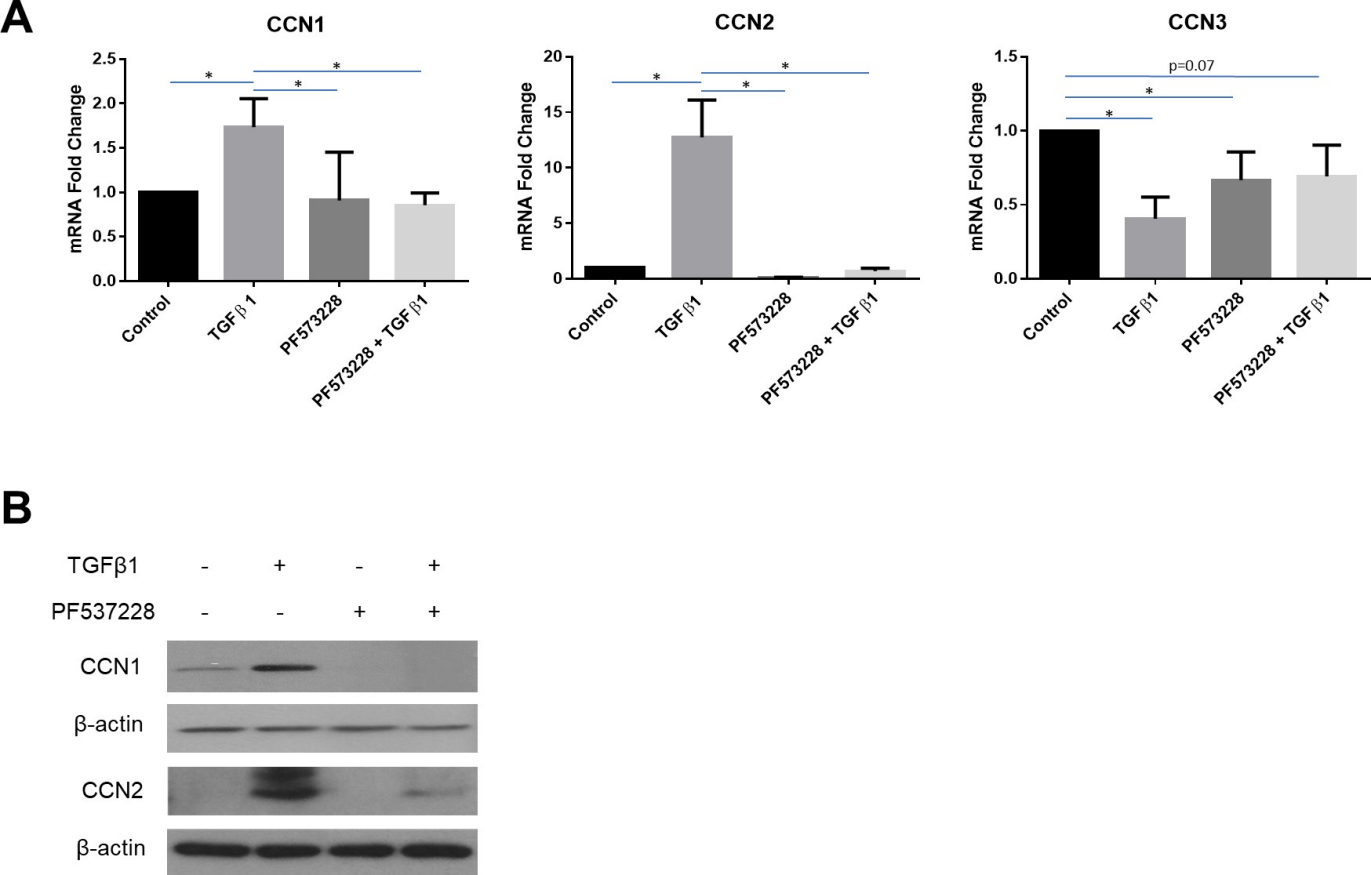
TGFbeta1-responsiveness of CCN1 and CCN2 is sensitive to FAK inhibition in human dermal fibroblasts. Human dermal fibroblasts were cultured, and serum starved overnight, prior to pre-treatment with a FAK inhibitor, PF573228 (10 μM). After pre-treatment, cells were incubated either with or without TGFbeta1 (4 ng/ml). **(A)** Total RNA was extracted 6 hours after treatment and subjected to real-time PCR to detect changes in CCN1, CCN2 and CCN3 gene expression. Results are expressed as mean +/- SD. Statistical differences were determined using one-way ANOVA with Tukey’s post-hoc test (p<0.05, n = 4). **(B)** Protein was extracted 24 hours after treatment and equal amounts of protein (50 μg) were resolved using SDS-PAGE. Western blot analysis was performed using antibodies against CCN1 and CCN2. Beta-actin was used as a loading control. Representative blots are shown (n = 3).

Prior data from our group and others has suggested that an autocrine proadhesive signaling loop operating through focal adhesion kinase (FAK) sustains fibrosis, and FAK appears to mediate TGFbeta’s profibrotic effects [11, 39, 40]. To extend our current data, we then assessed whether addition of a FAK inhibitor could affect the ability of TGFbeta1 to modulate mRNA expression of CCN1, CCN2, and CCN3 in human dermal fibroblasts. We conducted experiments similar to those described above, however we cultured cells in the presence or absence of TGFbeta1 and the presence or absence of the FAK inhibitor PF573228. We found that PF573228 blocked TGFbeta1-induced CCN1 and CCN2 mRNA and protein expression in human dermal fibroblasts (Fig 2). TGFbeta1-suppressed CCN3 expression was insensitive to PF573228 (Fig 2). Given the profibrotic roles of CCN2 and CCN1, these results emphasize the critical, central role of adhesive signaling operating through FAK in mediating fibrogenic responses in response to TGFbeta1.

### TGFbeta1 induces CCN1 and CCN2 yet suppresses CCN3 expression in human dermal fibroblasts via MEK

**Fig 3:**
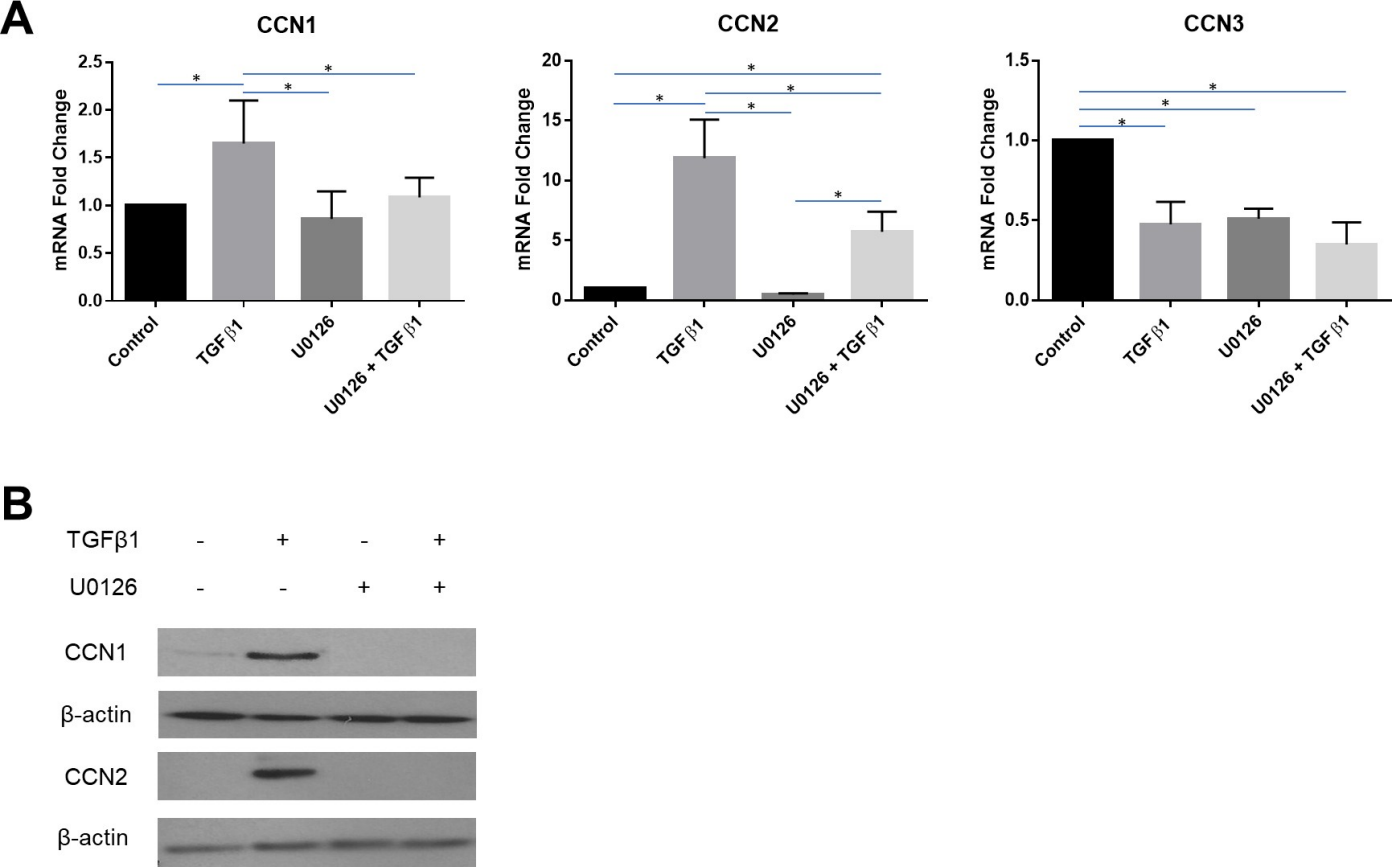
TGFbeta1-responsiveness of CCN1 and CCN2 is sensitive to MEK inhibition in human dermal fibroblasts. Human dermal fibroblasts were cultured, and serum starved overnight, prior to pre-treatment with a MEK inhibitor, U0126 (30 μM). After pre-treatment, cells were incubated either with or without TGFbeta1 (4 ng/ml). **(A)** Total RNA was extracted 6 hours after treatment and subjected to real-time PCR to detect changes in CCN1, CCN2 and CCN3 gene expression. Results are expressed as mean +/- SD. Statistical differences were determined using one-way ANOVA with Tukey’s post-hoc test (p<0.05, n = 3). **(B)** Protein was extracted 24 hours after treatment and equal amounts of protein (50 μg) were resolved using SDS-PAGE. Western blot analysis was performed using antibodies against CCN1 and CCN2. Beta-actin was used as a loading control. Representative blots are shown (n = 3).

Prior data from our group has indicated that TGFbeta-induced CCN2 expression occurs via MEK/ERK [41]. To assess if the ability of TGFbeta1 to induce CCN1 mRNA and protein expression and suppress CCN3 mRNA expression depended on MEK, we repeated our studies in the presence or absence of the MEK inhibitor U0126. As anticipated, consistent with our prior reports, TGFbeta1-induced CCN2 mRNA and protein expression in a manner that was impaired by U0126 [41] (Fig 3). Similarly, addition of U0126 significantly impaired the ability of TGFbeta1 to induce CCN1 mRNA and protein expression in human dermal fibroblasts (Fig 3). Finally, addition of U0126 did not significantly impaired the ability of TGFbeta1 to suppress CCN3 mRNA expression in human dermal fibroblasts, although U0126 reduced baseline CCN3 mRNA expression (Fig 3). These data emphasize the central role of MEK in mediating the fibrogenic responses of TGFbeta1.

### TGFbeta1 induces CCN1 and CCN2 in human dermal fibroblasts via TAK1

Mitogen-activated protein kinase kinase kinase 7 (MAP3K7), also known as TAK1 (TGFbeta-activated kinase 1), once activated, is an upstream activator of MKK/JNK and p38 by the phosphorylation and activation of MAP kinase kinases such as MAP2K1/MEK1, MAP2K3/MKK3, MAP2K6/MKK6 and MAP2K7/MKK7. TAK1 deletion blocks TGFbeta-induced alpha-smooth muscle actin expression in mouse embryonic fibroblasts, and, in gingival fibroblasts, the TAK1 inhibitor (5Z)-7-Oxozeaenol blocks the ability of TGFbeta1 to induce CCN2 expression [42,43]. To extend these studies, we assessed the ability of (5Z)-7-Oxozeaenol to block the effect of TGFbeta1 on CCN1, CCN2 and CCN3 expression in human dermal fibroblasts.

**Fig 4:**
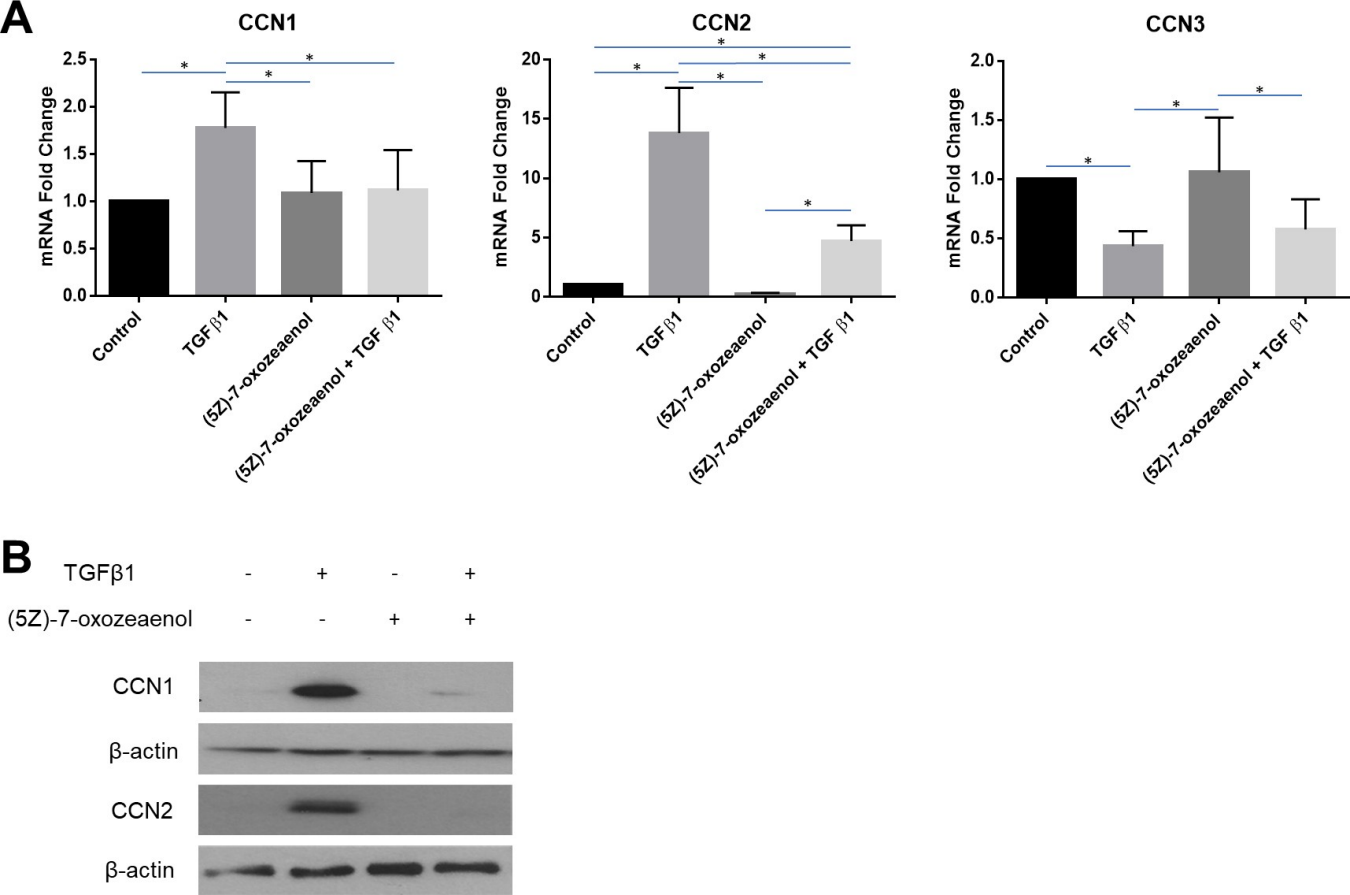
TGFbeta1-responsiveness of CCN1 and CCN2, but not CCN3, is sensitive to TAK inhibition in human dermal fibroblasts. Human dermal fibroblasts were cultured, and serum starved overnight, prior to pre-treatment with a TAK1 inhibitor, (5Z)-7-Oxozeaenol (400 μM). After pre-treatment, cells were incubated either with or without TGFbeta1 (4 ng/ml). **(A)** Total RNA was extracted 6 hours after treatment and subjected to real-time PCR to detect changes in CCN1, CCN2 and CCN3 gene expression. Results are expressed as mean +/- SD. Statistical differences were determined using one-way ANOVA with Tukey’s post-hoc test (p<0.05, n = 6). **(B)** Protein was extracted 24 hours after treatment and equal amounts of protein (50 μg) were resolved using SDS-PAGE. Western blot analysis was performed using antibodies against CCN1 and CCN2. Beta-actin was used as a loading control. Representative blots are shown (n = 3).

We found that, when applied to human dermal fibroblasts, (5Z)-7-Oxozeaenol treatment impairedTGFbeta1-induced CCN1 and CCN2 mRNA and protein expression; however, TGFbeta1-suppressed CCN3 mRNA expression was not significantly affected by (5Z)-7-Oxozeaenol (Fig 4). These data suggest that the mechanism by which TGFbeta1 induces CCN2 and CCN1 is divergent from that mediating TGFbeta1-suppressed CCN3 mRNA expression.

### TGFbeta1 induces CCN1 and CCN2 in human dermal fibroblasts via YAP1

Previously, we showed that CCN2 expression was promoted by the mechanosensitive oncogene YAP1 [41]. Subsequently, the literature has used both CCN1 and CCN2 as stereotypical genes induced by the hippo/YAP/TAZ pathway [44]. Given these observations and the potential importance of mechanotransduction in mediating pathological fibrosis, we ascertained if the selective YAP1 inhibitor verteporfin [45] could impair the effect of TGFbeta1 on CCN1, CCN2 and CCN3 expression in human dermal fibroblasts.

**Fig 5:**
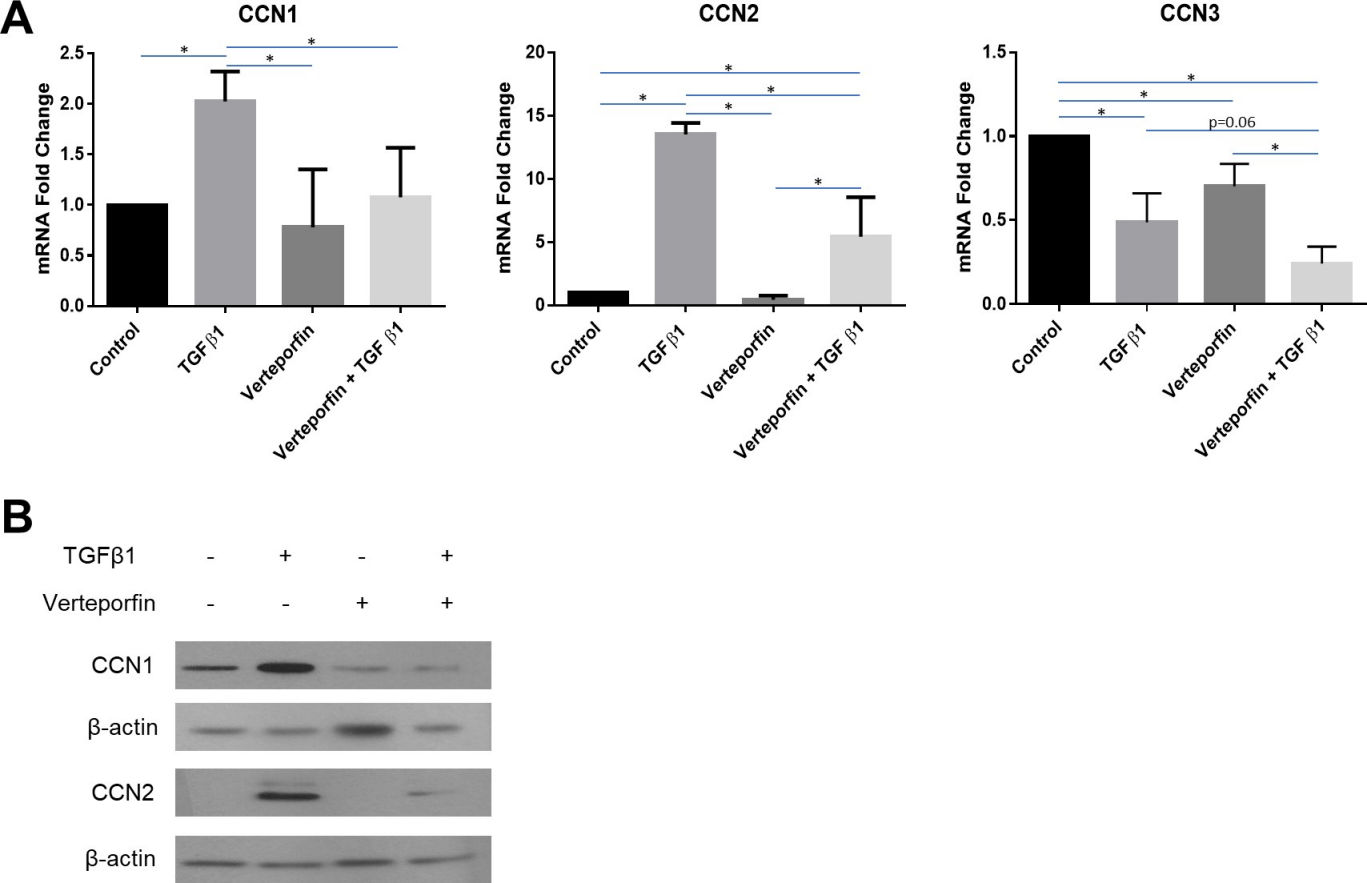
TGFbeta1-responsiveness of CCN1 and CCN2, but not CCN3, is sensitive to YAP inhibition in human dermal fibroblasts. Human dermal fibroblasts were cultured, and serum starved overnight, prior to pre-treatment with the YAP inhibitor verteporfin (295 μM). After pre-treatment, cells were incubated either with or without TGFbeta1 (4 ng/ml). **(A)** Total RNA was extracted 6 hours after treatment and subjected to real-time PCR to detect changes in CCN1, CCN2 and CCN3 gene expression. Results are expressed as mean +/- SD. Statistical differences were determined using one-way ANOVA with Tukey’s post-hoc test (p<0.05, n = 6). **(B)** Protein was extracted 24 hours after treatment and equal amounts of protein (50 μg) were resolved using SDS-PAGE. Western blot analysis was performed using antibodies against CCN1 and CCN2. Beta-actin was used as a loading control. Representative blots are shown (n = 3).

In human dermal fibroblasts, exposure to verteporfin impaired the ability of human dermal fibroblasts to respond to TGFbeta1 by increasing CCN1 and CCN2 mRNA and protein expression (Fig 5). However, YAP1 inhibition did not significantly affect the ability of TGFbeta1 to suppress CCN3 mRNA expression and, in fact, appeared to enhance the suppressive effect of TGFbeta in CCN3 mRNA expression (p = 0.06) (Fig 5). These data are consistent with the notions that verteporfin might be used to suppress the fibrogenic effects of TGFbeta1 on fibroblasts; and that the mechanism underlying the ability of TGFbeta1 to induce CCN2 and CCN1 differs from that through which TGFbeta1 suppresses CCN3 mRNA expression.

### Overexpression of CCN3 suppresses TGFbeta1-induced CCN2 protein expression

**Fig 6:**
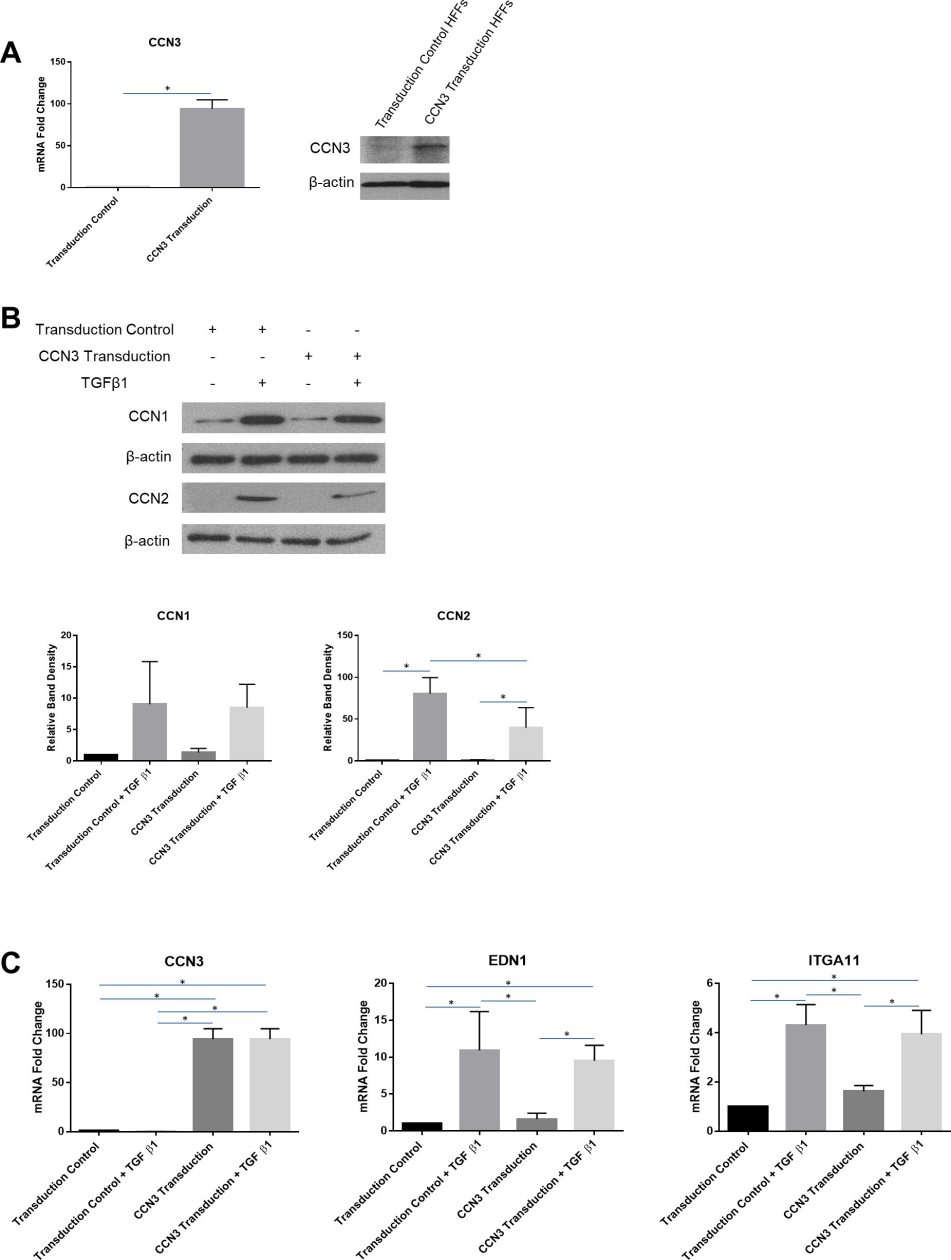
CCN3 overexpression impairs TGFbeta1-induced CCN2 protein expression. Human dermal fibroblasts were transduced with lentiviral particles containing a CCN3 expression vector, while transduction-control cells were transduced with lentiviral particles containing a control expression vector. **(A)** CCN3 overexpression was confirmed via qPCR and Western blot analysis. CCN3-transduced cells show significantly more CCN3 mRNA and protein expression than transduction-control cells. Statistical differences were determined using an unpaired t-test (p<0.05, n = 3). **(B)** Cells were serum-starved overnight and then treated with or without TGFbeta1 (4 ng/ml). Protein was extracted 24 hours after treatment and equal amounts of protein (50 μg) were resolved using SDS-PAGE. Western blot analysis was performed using antibodies against CCN1 and CCN2. Beta-actin was used as a loading control. Representative blots are shown (n = 3). Densitometry analysis was performed using ImageJ software and statistical differences were determined using one-way ANOVA with Tukey’s post-hoc test (p<0.05). **(C)** Total RNA was extracted after treatment with or without TGFbeta (4 ng/ml) and subjected to real-time PCR to detect changes in CCN3, EDN1, and ITGA11. Results are expressed as mean +/- SD. Statistical differences were determined using one-way ANOVA with Tukey’s post-hoc test (p<0.05, n = 3).

CCN3 has been proposed to be a potential anti-fibrotic treatment [14]. To examine the possible mechanism underlying such an activity we generated human dermal fibroblasts overexpressing CCN3. In these cells, TGFbeta1 was able to induce CCN1 protein expression; however, the ability of TGFbeta1 to induce CCN2 protein was significantly impaired (Fig 6A and 6B). Unsurprisingly, as: (a) in transduced fibroblasts CCN3 was overexpressed under the control of the non-TGFbeta-responsive CMV promoter; and (b) loss of CCN2 does not impair the ability of TGFbeta to induce expression of fibrogenic mRNA in cultured dermal fibroblasts [18], overexpression of CCN3 was not (a) suppressed by the addition of TGFbeta1 (b) able to impair the ability of TGFbeta to induce the mRNA expression of the fibrogenic markers endothelin-1 (EDN1) or integrin alpha 11 (ITGA11) [46–48] (Fig 6C).

## Discussion

The connective tissue microenvironment is being increasingly appreciated in playing a central role in disease [2,49]. Specifically, the CCN family of matricellular proteins show altered expression patterns in connective tissue disease and are emerging targets for therapeutic intervention [50]. CCN proteins share a similar structure, and have limited in vitro effects, making the development of relevant cell-based bioassays extremely difficult; consequently, is is necessary to study the functional role of CCN proteins in vivo. [15, 30] Of these, CCN2 (CTGF) is the most studied; indeed, anti-CCN2 antibodies are in clinical development [23]. Other CCN family members may have different functional roles in vivo, and indeed, may have opposing effects [30]. Of the other CCN proteins, CCN1 and CCN3 are the most studied; data published thus far suggest that, in vivo, CCN1 may have context-dependent profibrotic effects, whereas CCN3 may be antifibrotic [25–29]. However, until now, no study has simultaneously compared the expression of CCN1, CCN2 and CCN3 in response to the profibrotic protein TGFbeta1 in cultured dermal fibroblasts. Our data suggest that CCN1 and CCN2 are induced by TGFbeta1 via a similar pathway; conversely CCN3 mRNA is reduced by TGFbeta1 through a pathway that is divergent; that is, not involving MEK1, YAP1 or TAK1. These data are consistent with the general hypothesis that CCN1 and CCN2 are expressed in a yin/yang fashion in a way opposite to CCN3 and with a hypothesis that, in vivo, restoring a CCN1/2:CCN3 balance may be of therapeutic value [14, 30]. Similar to previous data showing that CCN2-deficient dermal fibroblasts retained TGFbeta-responsiveness [18], overexpressing CCN3 in human dermal fibroblasts had no appreciable effect on the ability of TGFbeta to induce mRNA expression of endothelin-1 or integrin alpha 11. It should be reiterated that loss or blockade of CCN2 expression severely impairs fibrogenesis including myofibroblast differentiation in vivo in a fashion that does not appear to involve canonical TGFbeta signaling [15, 18–21, 51,52]. Since CCN proteins have limited ex vivo effects and instead act to integrate signaling emanating from multiple sources [30], direct testing of the potential antifibrotic role of CCN3 requires the use of animal models and is therefore beyond the scope of our current report.

Our studies investigating the mechanism of how non-canonical TGFbeta signaling activates CCN1 and CCN2 expression support the notion that FAK, ERK, TAK1 and YAP1 promote fibrogenic responses. The involvement of adhesive signaling via FAK and ERK [2, 40, 53] in promoting TGFbeta signaling and fibrosis is consistent with prior reports in other systems. YAP1 is known to activate genes in response to mechanotransduction; however, relatively few reports have examined the effect of verteporfin in blocking TGFbeta’s fibrogenic responses. Indeed, only two other reports have examined this question. Specifically, verteporfin was shown to reduce TGFbeta responses in NRK renal cells and in conjunctival fibroblasts [54, 55]. This result is of potential long-term clinical application as verteporfin is in clinical use for macular degeneration [56].

Collectively, our data provide new and valuable insights into the coordinated and opposite regulation of the key CCN family members CCN1, CCN2 and CCN3 in human dermal fibroblasts and are consistent with the long-term hypothesis that alterations in CCN1/CCN2:CCN3 ratio in response to fibrogenic stimuli may be important in driving fibrogenic responses. Our results showing that overexpression of CCN3 in fibroblasts reduces the ability of TGFbeta to induce CCN2 protein expression are consistent with that notion. Our results are also consistent with the notion that TGFbeta induces fibrogenic responses in fibroblasts via non-canonical proadhesive/mechanotransductive pathways and that targeting this pathway, e.g. by verteporfin, may be of value.
